# Patients with cognitive deficits and substance use disorders, a clinical population in need of focused attention

**DOI:** 10.3389/fpsyt.2023.1281914

**Published:** 2023-10-12

**Authors:** Aleksander H. Erga, Jens Hetland, Kirsten Braatveit

**Affiliations:** ^1^Center for Alcohol and Drug Research (KORFOR), Stavanger University Hospital, Stavanger, Norway; ^2^The Norwegian Centre for Movement Disorders, Stavanger University Hospital, Stavanger, Norway; ^3^Department of Social Studies, University of Stavanger, Stavanger, Norway; ^4^Department of Biological and Medical Psychology, University of Bergen, Bergen, Norway; ^5^Department of Research and Innovation, Helse Fonna HF, Haugesund, Norway

**Keywords:** addiction, cognition, intellectual functioning, substance use disorder, treatment

## Introduction

Through multidisciplinary research efforts, our understanding of substance use disorders (SUDs) is ever increasing. In a recent review by Volkow and Blanko, the importance of addressing the needs of special populations in SUD-research was emphasized (1). These populations warrant special attention in both clinical settings and future research. This focus is welcomed and highlights the move toward more individualized, tailored, and personalized treatment approaches (2). A personalized treatment approach for SUDs depends on detailed knowledge of how biological, psychological, and social factors impact the severity, trajectory, and recovery of SUDs (3–5). Thus, targeted efforts are needed to enable personalized treatment approaches for SUD populations with distinct clinical needs (5). Indeed, these groups often experience adverse clinical outcomes and respond less to preventative strategies, which underscores the importance of future research efforts in this area.

## Cognitive dysfunction in SUD

One population that warrants special attention is patients with cognitive dysfunction (6). Cognitive impairments negatively impact treatment processes and therapeutic change mechanisms at all stages of SUD treatment. Further, within this group, reduced insight and recognition of problem use impede efficient prevention and early identification of SUDs (7, 8), and difficulties with adherence and attendance are frequent in outpatient, inpatient, and aftercare treatment settings (9, 10). Moreover, standard treatment approaches are often unsuitable and contribute to elevated dropout and relapse rates among patients with cognitive impairment (11–13). Indeed, patients with cognitive impairment may follow different recovery pathways compared to those without such impairments. For instance, informal treatment processes and social structures may play a more prominent role in determining behavioral, psychosocial, emotional, and vocational outcomes (9, 14).

The advances in understanding the neurobiology of SUDs, have highlighted the importance of translating basic research findings into implementable therapeutic interventions. Cognitive dysfunction, such as increased impulsivity, altered decision making, and impaired executive functioning in the early stage of drug abstinence, is well-documented in SUDs (6). However, cognitive impairments in SUDs are not limited to transient cognitive alterations attributable to prolonged drug use or the effects of acute withdrawal. Enduring cognitive impairment can be related to a host of non-drug factors, including head trauma, co-morbid psychiatric disorders, cerebrovascular changes, and congenital or premorbid cognitive impairment (15–17). In the clinical research pipeline, novel treatment options such as contingency management and cognitive remediation therapies hold promise as personalized and viable strategies for managing cognitive impairments in the SUD population. Indeed, these novel therapeutic advances could serve as valuable adjunctive treatments to standard SUD-treatment, like cognitive behavioral therapy, in cases where delayed discounting or reward based decision-making is prominent in the patients case formulation (18). In therapeutic community treatment, adjunctive group based cognitive remediation therapy, also shows promising findings (19). The development of treatment algorithms that assist clinicians and patients in the choice of treatment modality will be essential for efficient and personalized implementation these treatment approaches (20).

There is a growing recognition that patients with SUDs and mild to borderline intellectual disability (MBID) (IQ 50–85) represent a special clinical population that needs dedicated and targeted clinical and research attention. First, individuals with MBID may have an augmented vulnerability to the development of SUDs compared to those without these intellectual deficits (2, 21). In addition, they may experience more severe mental, physical, and social consequences from substance use than members of other groups (2). Second, individuals with impaired intellectual functioning are likely to experience barriers to accessing substance abuse treatment (22, 23). Third, although traditionally identified and treated in intellectual disability (ID) services, recent studies indicate a large unidentified population of patients with premorbid intellectual impairment in facilities offering treatment for SUD with prevalence rates reaching up to 39% (12, 21, 24). The under-recognition of comorbid SUD and intellectual impairment, coupled with the inherent difficulties in evaluating cognition and adaptive functioning among patients in the SUD population, may play a pivotal role in concealing this population (12, 25). Fourth, it is suggested that patients with intellectual deficits may not benefit from mainstream SUD treatment in its current form due to difficulties such as limited attention span, limited vocabulary, short and/or long-term memory problems, difficulties discriminating between relevant and irrelevant information, problems with planning and attention, impaired abstract reasoning, and low self-insight (26). Moreover, compared to individuals with mental health issues in the general population, they are also more likely to receive mono-therapy, which limits these patients in their recovery from SUDs and their integration into society as fully functional citizens (27, 28).

## Discussion

The comorbidity of SUD and MBID has largely been overlooked in the addiction medicine literature (21). We argue that recognizing and adopting a personalized treatment approach for patients with premorbid intellectual impairments is essential to improve treatment outcomes for individuals with SUDs. However, this approach is not without its challenges. The primary organizational obstacle is how to provide ongoing care for this group. Patients with both SUD and intellectual deficits will require continuous community care that extends beyond standard inpatient or outpatient services. Most countries have dedicated ID services for individuals with ID, and there is a need for collaboration and knowledge exchange between ID and SUD services (21, 29). Although found to struggle in a number of life and personal areas (30), most countries lack dedicated services targeting the intellectual impairment associated with borderline intellectual functioning (IQ 70–85). These patients are frequently deemed unsuitable for specialized services, leading to their displacement across various service providers and a consequent lack of adequate support.

While advances in screening and assessment of cognitive functioning in patients with SUD will result in increased identification of MBID and other cognitive dysfunctions (31), the development of novel inpatient, outpatient, and community prevention and care strategies for these patients is needed. In the years to come, it is crucial we identify barriers for treatment adherence, algorithms in how to tailor psychosocial interventions, and systems to assist patients benefiting from pharmaceutical treatments. Indeed, we share Volow and Blancos optimism that focused research will generate novel and more effective therapeutic interventions that are tailor for the individual patient (1).

## Author contributions

AE: Conceptualization, Writing—original draft, Writing—review and editing. JH: Writing—original draft, Writing—review and editing. KB: Writing—original draft, Writing—review and editing.
